# Efficient detoxication of hydroxylamine and nitrite through heterotrophic nitrification and aerobic denitrification by *Acinetobacter johnsonii* EN-J1

**DOI:** 10.3389/fmicb.2023.1130512

**Published:** 2023-04-17

**Authors:** Manman Zhang, Tengxia He, Qifeng Wu, Mengping Chen

**Affiliations:** Key Laboratory of Plant Resource Conservation and Germplasm Innovation in Mountainous Region (Ministry of Education), Collaborative Innovation Center for Mountain Ecology Agro-Bioengineering (CICMEAB), College of Life Sciences, Institute of Agro-Bioengineering, Guizhou University, Guiyang, Guizhou, China

**Keywords:** nitrogen removal rate, HN-AD, enzyme activities, nitrogen balance, hydroxylamine and nitrite

## Abstract

The co-existence of hydroxylamine (NH_2_OH) and nitrite (NO_2_^–^-N) can aggravate the difficulty of wastewater treatment. The roles of hydroxylamine (NH_2_OH) and nitrite (NO_2_^–^-N) in accelerating the elimination of multiple nitrogen sources by a novel isolated strain of *Acinetobacter johnsonii* EN-J1 were investigated in this study. The results demonstrated that strain EN-J1 could eliminate 100.00% of NH_2_OH (22.73 mg/L) and 90.09% of NO_2_^–^-N (55.32 mg/L), with maximum consumption rates of 1.22 and 6.75 mg/L/h, respectively. Prominently, the toxic substances NH_2_OH and NO_2_^–^-N could both facilitate nitrogen removal rates. Compared with the control treatment, the elimination rates of nitrate (NO_3_^–^-N) and NO_2_^–^-N were enhanced by 3.44 and 2.36 mg/L/h after supplementation with 10.00 mg/L NH_2_OH, and those of ammonium (NH_4_^+^-N) and NO_3_^–^-N were improved by 0.65 and 1.00 mg/L/h after the addition of 50.00 mg/L NO_2_^–^-N. Furthermore, the nitrogen balance results indicated that over 55.00% of the initial total nitrogen was transformed into gaseous nitrogen by heterotrophic nitrification and aerobic denitrification (HN-AD). Ammonia monooxygenase (AMO), hydroxylamine oxidoreductase (HAO), nitrate reductase (NR), and nitrite reductase (NIR), which are essential for HN-AD, were detected at levels of 0.54, 0.15, 0.14, and 0.01 U/mg protein, respectively. All findings confirmed that strain EN-J1 could efficiently execute HN-AD, detoxify NH_2_OH and NO_2_^–^-N, and ultimately promote nitrogen removal rates.

## 1. Introduction

During the nitrogen biodegradation process, hydroxylamine (NH_2_OH) and nitrite (NO_2_^–^-N), as intermediate metabolites of heterotrophic nitrification and aerobic denitrification (HN-AD), are toxic to most nitrogen-transforming bacteria. NH_2_OH can destroy the protein translation process involved in the bacterial ribosome so that bacteria cannot reproduce and grow, thus affecting the nitrogen removal ability of the bacteria (Xing et al., 2020). NO_2_^–^-N has been described as an inhibitor of bacterial activity with known toxicity (Baumann et al., 1997). For example, NO_2_^–^-N could inhibit oxygen uptake, oxidative phosphorylation, and active transport of glucose and proline in *P. aeruginosa* (Yarbrough et al., 1980). The mRNA level of amoA (ammonia monooxygenase) in *N. multiformis* was significantly inhibited by 20 mM NO_2_^–^-N, which further affected the nitrogen conversion ability of the strain (Cua and Stein, 2011). The accumulation of NH_2_OH and NO_2_^–^-N can also lower the total nitrogen (TN) conversion efficiency (Ouyang et al., 2020). Therefore, it is necessary to isolate and identify HN-AD strains that can efficiently and simultaneously convert NH_2_OH and NO_2_^–^-N. Although multiple HN-AD, simultaneous nitrification and denitrification (SND) and aerobic denitrification bacteria have been reported and characterized by sustainable growth and low energy consumption, the conversion rates of inorganic nitrogen and TN were universally low, especially for the toxic nitrogen sources of NH_2_OH and NO_2_^–^-N. For example, the NO_2_^–^-N removal rates of *Bacillus methylotrophicus* L7, *Pannonibacter phragmitetus* B1 and *Acinetobacter tandoii* MZ-5 were only 0.24, 0.81, and 1.18 mg/L/h, respectively, (Ren et al., 2014; Bai et al., 2019; Ouyang et al., 2020). *Alcaligenes faecalis* No. 4 was confirmed to have the ability to degrade NH_2_OH with a conversion rate of 4.70 mg/L/h, but it could not remove NO_2_^–^-N (Joo et al., 2005). Although NH_2_OH and NO_2_^–^-N could be oxidized and reduced by *Pseudomonas taiwanensis* J488, their conversion rates were as low as 0.80 and 1.28 mg/L/h, respectively, (He et al., 2020). Analogously, the actinomycete *Streptomyces mediolani* EM-B2 was found to be capable of NH_2_OH and NO_2_^–^-N degradation, but the corresponding maximum consumption rates were only 0.43 and 2.01 mg/L/h, respectively, (He et al., 2021). Furthermore, numerous bacteria cannot simultaneously remove several nitrogen sources when NH_2_OH or NO_2_^–^-N coexist in the culture.

To date, most of studies have reported the effects of NH_2_OH and NO_2_^–^-N on the HN-AD process to convert nitrogen. For example, a concentration of NH_2_OH between 7.50 and 35.0 mg/L could delay NO_2_^–^-N reduction (Noophan et al., 2004). When the NH_2_OH level exceeded 35.00 mg/L, the cytotoxicity of NH_2_OH could reduce the activities of denitrifying bacteria and further lower the nitrate (NO_3_^–^-N) degradation rate (Zhang et al., 2020). Several reports indicated that the presence of NH_2_OH could promote bacterial abundance and the generation of nitrogen removal products. For instance, the abundance of ammonia-oxidizing bacteria was significantly enhanced when 5.00 mg/L NH_2_OH was supplemented (Yu et al., 2019). The accumulation of gaseous nitrogen in the form of NO was improved (0 → 0.23 mg/L) by the continuous addition of 5.00 mg/L NH_2_OH (Zhao et al., 2021). However, most reports on the effects of hydroxylamine on biological nitrogen removal have mainly focused on activated sludge, biofilms, and sequencing batch reactors, which are subject to various environmental factors and could not accurately assess the effects of hydroxylamine on biological nitrogen removal. Regarding the influence of NO_2_^–^-N, current reports have mainly focused on its negative effect on the HN-AD process. For instance, He et al. (2017) confirmed that supplementation with a low dosage of NO_2_^–^-N (5.33 mg/L) exhibited an inhibitory effect on nitrification and the ammonium (NH_4_^+^-N) conversion rate decreased from 0.40 mg/L/h to 0.12 mg/L/h at 15°C. Bettazzi et al. (2010) found that supplementation with 75.00 mg/L NO_2_^–^-N lowered anammox activity. However, positive effects of NO_2_^–^-N on HN-AD have not yet been reported. In addition, the influence of NH_2_OH and NO_2_^–^-N on the conversion rates of diverse inorganic nitrogen species has not yet been thoroughly studied, which limits the comprehensive evaluation of bacterial nitrogen removal ability in wastewater treatment. Therefore, identifying bacteria that can rapidly transform the toxic nitrogen sources NH_2_OH and NO_2_^–^-N without restricting the degradation rates of multiple inorganic nitrogen forms, may enhance wastewater treatment performance.

A previous report found that *A. johnsonii* could perform aerobic denitrification and that the lower-molecular-weight organic matter could promote the cell growth of *A. johnsonii* during the NO_3_^–^-N conversion process (Wen et al., 2019). Moreover, *A. johnsonii* exhibited outstanding partial denitrification performance and the key functional genes *napA, nirB*, and *nirD* were expressed under optimal environmental conditions (Zhang et al., 2019). However, the NH_2_OH and NO_2_^–^-N nitrogen conversion capabilities by *A. johnsonii* and the effects on HN-AD have not been reported. Therefore, the objectives of this study were to (1) focus on the isolation of an efficient HN-AD strain that can rapidly transform the toxic nitrogen sources NO_2_^–^-N and NH_2_OH and then identify it based on its morphology and molecular characteristics; (2) explore the HN-AD capacity in the presence of different nitrogen forms [(NH_4_)_2_SO_4_, KNO_3_, NaNO_2_, and HONH_3_Cl]; (3) investigate the effects of external NH_2_OH addition on the HN-AD process and cell growth by using batch tests; (4) examine the effects of NO_2_^–^-N on the HN-AD process and cell growth; and (5) elucidate the HN-AD mechanism through nitrogen balance analysis and the detection of related enzyme expression. The experimental results showed that strain EN-J1 could efficiently execute HN-AD, detoxify NH_2_OH and NO_2_^–^-N, and promote the nitrogen conversion rates and cell growth; these findings provide a theoretical and experimental basis for enhancing nitrogen conversion rates in wastewater pollution control.

## 2. Materials and methods

### 2.1. Bacterial isolation

The samples used in this experiment were taken from towel gourd vegetable cultivation soil (5∼10 cm depth) from Jinping County, southeastern Guizhou. For preliminary screening of the strains with the highest HN-AD ability, 1 g of soil was added to the enrichment medium, which consisted of (per liter) HONH_3_Cl (0.05 g), NaNO_2_ (0.10 g), C_6_H_5_Na_3_O_7_.2H_2_O (1.84 g), K_2_HPO_4_ (3.50 g), MgSO_4_ (0.04 g), CaCI_2_ (0.01 g), Fe_2_(SO_4_)_3_ (0.01 g), KH_2_PO_4_ (1.50 g), pH = 7.20 and transfer was performed 3 times at 4-day intervals (150 rpm, 25°C). The enriched medium (5, 10, 15 μL) was extracted and then purified on bromothymol blue (BTB) solid medium, which was made with (per liter, pH = 7.00) 1 mL BTB reagent (1.50% in ethanol), (NH_4_)_2_SO_4_ (0.24 g), sodium citrate (2.45 g), FeSO_4_.7H_2_O (0.59 g), KH_2_PO_4_ (1.00 g), CaCl_2_ (0.09 g), MgSO_4_ (0.49 g), and agar (18 g) (He et al., 2018). Bacteria with blue colonies and the highest removal rates of NH_2_OH and NO_2_^–^-N were selected as the candidate strain and stored at −20°C (30% glycerol). The basic medium composition for assessment of the nitrogen removal capability by the candidate contained (per liter, pH = 7.20) KH_2_PO_4_ (1.50 g), K_2_HPO_4_ (3.50 g), C_6_H_5_Na_3_O_7_.2H_2_O (3.06 g), Fe_2_(SO_4_)_3_ (0.01 g), MgSO_4_ (0.04 g), and CaCI_2_ (0.01 g) (He et al., 2019). (NH_4_)_2_SO_4_ (0.24 g), HONH_3_Cl (0.10 g), KNO_3_ (0.36 g), and NaNO_2_ (0.25 g) were added separately to the basic medium to analyze the nitrogen conversion ability of the target strain. The content of the carbon source was changed to 1.23 g C_6_H_5_Na_3_O_7_.2H_2_O with NH_2_OH only as the nitrogen source. To study the effects of NH_2_OH on HN-AD, the medium was modified to contain (per liter, pH = 7.20) HONH_3_Cl (0.05 g), KNO_3_ (0.36 g), NaNO_2_ (0.25 g) or (NH_4_)_2_SO_4_ (0.24 g), MgSO_4_ (0.04 g), K_2_HPO_4_ (3.50 g), CaCl_2_ (0.01 g), Fe_2_(SO_4_)_3_ (0.01 g), KH_2_PO_4_ (1.50 g), and C_6_H_5_Na_3_O_7_.2H_2_O (3.68 g). Similarly, the medium composition NaNO_2_ (0.25 g), (NH_4_)_2_SO_4_ (0.24 g) or KNO_3_ (0.36 g), MgSO_4_ (0.04 g), K_2_HPO_4_ (3.50 g), CaCl_2_ (0.01 g), KH_2_PO_4_ (1.50 g), Fe_2_(SO_4_)_3_ (0.01 g), and C_6_H_5_Na_3_O_7_.2H_2_O (6.13 g) was applied to study the influence of NO_2_^–^-N on HN-AD. Luria-Bertani (LB) medium containing tryptone (10.00 g), NaCl (10.00 g) and yeast extract (5.00 g) was used for bacterial activation and cultivation before all simulated wastewater experiments. All media were sterilized for 30 min (121°C, 0.11 MPa).

### 2.2. Molecular identification

Bacterial suspensions of strain EN-J1 were coated on LB plates and cultivated at 25°C until colony formation. Colony morphologies were observed *via* an SU8100 scanning electron microscope (Beijing, China). Gram staining of the bacterial cells was assessed with an Olympus BX53-DIC optical microscope (Beijing, China). A DNA extraction kit (Magen) was applied to extract genomic DNA of strain EN-J1 that was selected as a template to amplify 16S rRNA gene sequences. Amplification of the 16S rRNA gene by polymerase chain reaction (PCR) (25 μL reaction system) was conducted with the universal primers 27F and 1492R by Bio-Rad (CFXConnect&T100, America) (He and Li, 2015), and the PCR products were detected by Sangon Biotech (Shanghai, China). Afterward, the 16S rRNA gene sequence returned from Sangon Biotech was submitted to the NCBI to obtain accession number. After alignment by BLAST, the phylogenetic tree reflecting the kinship of strain EN-J1 was founded by MEGA 7.0.

### 2.3. Evaluation of HN-AD capacity and the effects of NH_2_OH and NO_2_^–^-N on HN-AD

To evaluate the HN-AD capability of the selected strain EN-J1, the bacteria (glycerol-preserved) were inoculated in LB medium, cultivated at 25°C for 24 h with a 150 rpm shaking speed, and then harvested under centrifugation conditions (6,500 rpm, 25°C, 5 min). Strain EN-J1 was washed 3 times with sterile pure water to obtain a pure bacterial suspension. Bacteria with an optical density (OD_600_) value of 0.20 were inoculated into 100 mL media containing (approximately 50 mg/L) NH_4_^+^-N, NO_3_^–^-N or NO_2_^–^-N, and bacteria with an OD_600_ of 0.50 were added to 20 mg/L NH_2_OH medium, in which sodium citrate was applied as an electron donor. The procedure for studying the effects of NH_2_OH or NO_2_^–^-N on HN-AD was similar to that described above, except for the use of different carbon and nitrogen sources. Medium without bacterial addition was used as a control treatment. Six milliliters of medium supernatant was collected from the culture every 6 h to measure pH, OD_600_ and the concentrations of different inorganic nitrogen forms (including NH_4_^+^-N, NH_2_OH, NO_3_^–^-N, and NO_2_^–^-N) and TN. The conversion rates of inorganic nitrogen were calculated by the formula E_*v*_ = (C_1_-C_2_)/ΔT, in which E_*v*_ represents the nitrogen removal rate, and C_1_, C_2_ and ΔT signify the beginning and final nitrogen contents and time interval (Cheng et al., 2020). The results were used to further analyze the nitrogen conversion characteristics of strain EN-J1.

### 2.4. Nitrogen balance analysis

Bacterial solution cultivated in LB medium for 24 h at 25°C with a rotation speed of 150 rpm was separately inoculated into NH_4_^+^-N, NO_2_^–^-N, NH_2_OH, and NO_3_^–^-N and media. At the beginning of inoculation (0 h), the mixed bacterial solution (6 mL) was centrifuged at 6,500 rpm for 5 min, and then the supernatant was collected to detect the TN (initial TN1) and inorganic (NH_4_^+^-N, NH_2_OH, NO_2_^–^-N, and NO_3_^–^-N) concentrations. Simultaneously, another 6 mL of bacterial solution was harvested and lysed by ultrasonication for 15 min under 300 W power and a 3 s working/interval time with a Scientz-IID ultrasonicator. Afterward, a 0.22 mm filter membrane was used to filter the supernatant to detect the contents of NH_4_^+^-N, NH_2_OH, NO_3_^–^-N, and NO_2_^–^-N and final TN_1_. After cultivation in the NH_4_^+^-N, NO_3_^–^-N NH_2_OH, and NO_2_^–^-N media for 12, 18, 30, and 18 h, the new supernatants (6 mL) were collected, and the inorganic nitrogen (NH_4_^+^-N, NH_2_OH, NO_2_^–^-N, and NO_3_^–^-N) and initial TN_2_ were determined by repeating the above procedure. Another 6 mL bacterial solution was harvested and lysed, and the final NH_4_^+^-N, NH_2_OH, NO_3_^–^-N, NO_2_^–^-N and final TN_2_ were detected. All relevant nitrogen contents were calculated as follows (He et al., 2022):

(1) Organic N = initial TN_2_–final (NH_4_^+^-N + NH_2_OH + NO_2_^–^-N + NO_3_^–^-N); (2) Accumulated intracellular N = (final TN_2_ –initial TN_2_)–(final TN_1_–initial TN_1_); (3) Nitrogen loss (%) = [final TN_1_–final (NH_4_^+^-N + NH_2_OH + NO_2_^–^-N + NO_3_^–^-N)–Organic N–Accumulated intracellular N]/final TN_1_ × 100%.

### 2.5. Detection of HN-AD enzyme activities

The time with the highest nitrogen removal rate was selected to determine enzyme activity. After cultivation for 5, 21, 10, and 10 h with NH_4_^+^-N, NH_2_OH, NO_3_^–^-N, and NO_2_^–^-N media alone, strain EN-J1 was collected by centrifugation (6,500 rpm, 5 min, 25°C). Protein extraction in the crude extract of bacterial strain EN-J1 was performed using a BCA bacterial protein extraction kit (Solarbio, Beijing, China). The enzyme activity of ammonia monooxygenase (AMO) was determined by a testing company (Wela, Guiyang, China), and NO_2_^–^-N reductase (NIR) was detected by a NO_2_^–^-N reduction activity assay kit (COMIN, Suzhou Industrial Park, China). For NO_3_^–^-N reductase (NR) activity determination, the 20 mL reaction system contained NO_3_^–^-N, enzyme extract, NADH and Tris-HCl. The activity of NR was evaluated by the disappearance of NO_3_^–^-N after reaction for 15 min at 25°C (Zhang et al., 2022). In addition, a reaction mixture of 20 mL containing enzyme extract, NH_2_OH, potassium ferricyanide, EDTA and Tris-HCl was prepared for NH_2_OH oxidase (HAO) activity evaluation. The reduction in NH_2_OH content was taken as the measure to detect HAO activity. A control group was designed with no enzyme extract addition. The specific activity (U/mg) was calculated by the content of enzyme needed to degrade 1 μmol of substrate per minute divided by the protein concentration.

### 2.6. Analytical analysis

An ultraviolet (UV) spectrophotometer (Metash UV-6000) with a 600 nm wavelength was applied to monitor the bacterial solution concentration. After cell culture and centrifugation, the bacterial supernatant was used to evaluate the TN, NH_4_^+^-N, NO_2_^–^-N, NH_2_OH, and NO_3_^–^-N concentrations by alkaline potassium persulfate digestion-UV spectrophotometry, the indophenol blue method, 8-hydroxyquinoline UV spectrophotometry, UV spectrophotometry, and N-(1-naphthyl)-ethylenediamine photometry, respectively, (Chen et al., 2012). The pH meter of DDS-307A was applied to assess the pH value. All experimental data and figures were processed using SPSS Statistics, Excel, MEGA 7.0, and Origin2021 software and are presented as the mean ± SD.

## 3. Results

### 3.1. The isolated bacterium exhibited high NO_2_^–^-N and NH_2_OH removal capacities

**FIGURE 1:**
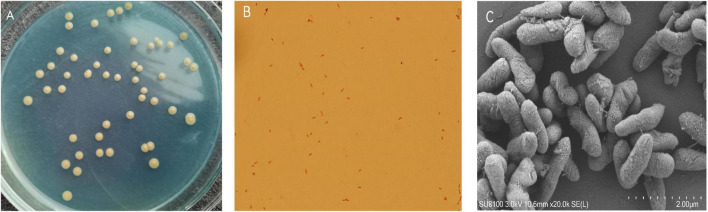
The colony morphologies of the strain EN-J1. **(A)** Colonies on BTB plate; **(B)** gram staining result under optical microscope; **(C)** cells shape by the scanning electron microscope.

**FIGURE 2:**
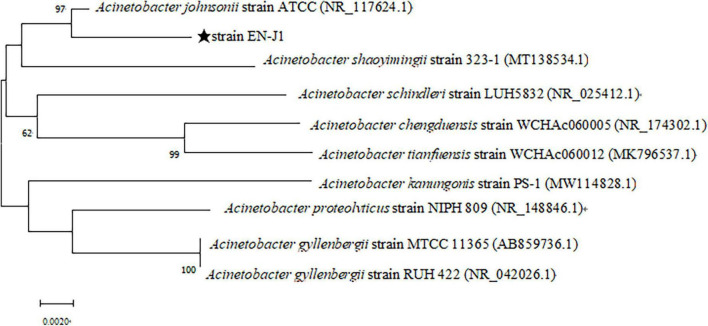
Neighbor-joining phylogenetic tree of the strain EN-J1 was structured based on its 16S rRNA gene sequence and other related strains sequences.

A mixed nitrogen source of NH_2_OH and NO_2_^–^-N was applied to obtain the HN-AD strain to increase the possibility of simultaneously removing multiple nitrogen sources. Thus, the newly screened strain EN-J1, which was isolated from a towel gourd vegetable cultivation field and purified on LB and BTB plates, exhibited outstanding NO_2_^–^-N and NH_2_OH removal ability. The colony morphological characteristics of strain EN-J1 were yellow, wet surface, regular edge, and convex (Figure 1A). Strain EN-J1 was found to be gram-negative by Gram staining and appeared short and rod-shaped without flagellum (Figures 1B, C); it was similar to strain *Acinetobacter* sp. HA2 except for colony color (Yao et al., 2013). The 16S rRNA nucleotide sequences were uploaded to GenBank under accession number ON076880. A phylogenetic tree demonstrating the kinship among related strains and strain EN-J1 was constructed with partial 16S rRNA sequences. Homology searches of strain EN-J1 *via* BLAST illustrated that it was highly similar (99.23%) to the gene sequences of *A. johnsonii* ATCC (Figure 2). To our knowledge, the nitrogen removal capability of NH_2_OH and NO_2_^–^-N by *A. johnsonii* and their effects on the HN-AD process have not been reported.

### 3.2. Characteristics of NH_4_^+^-N and NH_2_OH conversion

**FIGURE 3:**
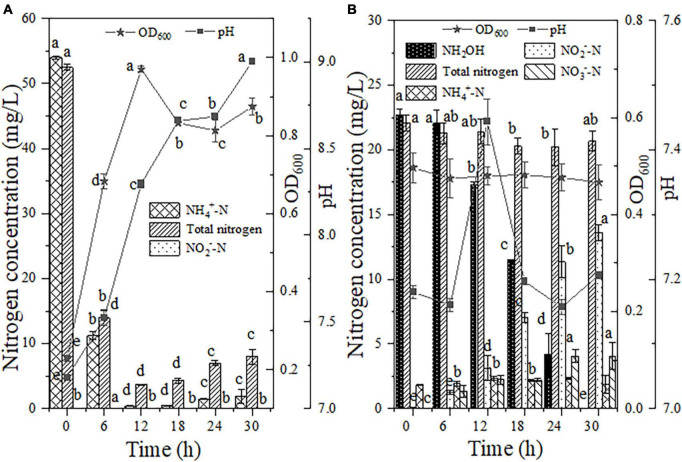
The heterotrophic nitrification characteristics of strain EN-J1. **(A)** Ammonium (NH_4_^+^-N); **(B)** hydroxylamine (NH_2_OH). Values are means ± SD. Different letters represent significant differences between treatments (*p* < 0.05).

The nitrification process was carried out with NH_4_^+^-N as the sole nitrogen source, and the nitrification traits of strain EN-J1 were analyzed on the basis of the removal of NH_4_^+^-N and the accumulation of intermediate products. Figure 3A shows that bacterial strain EN-J1 grew rapidly for the first 12 h after inoculation, and the OD_600_ value reached the highest level of 0.97. The NH_4_^+^-N content decreased significantly from 53.99 to 11.25 mg/L during the first 6 h, and 99.28% of NH_4_^+^-N was converted as the oxidation time was extended to 12 h. In the process of NH_4_^+^-N oxidation, the maximum degradation rate of 7.12 mg/L/h appeared during 0−6 h. Compared with previously reported NH_4_^+^-N oxidation strains, the rate of NH_4_^+^-N degradation was considerably greater than the values of 5.53 mg/L/h for *Pseudomonas stutzeri* YZN-001 (Xia et al., 2020), 3.70 mg/L/h for *Bacillus thuringiensis* WXN-23 (Xu et al., 2021), 3.46 mg/L/h for *Streptomyces mediolani* EM-B2 (He et al., 2022) and 2.29 mg/L/h for *Vibrio diabolicus* SF16 (Duan et al., 2015). Moreover, total nitrogen consumption reached 92.95%, corresponding to a maximum consumption rate of 6.42 mg/L/h, which indicated that much of the NH_4_^+^-N nitrogen was consumed and converted to gaseous nitrogen. After 18 h of continuous cultivation, the concentration of NH_4_^+^-N tended to increase based on the breakdown of dead cells (Li et al., 2015), which lowered the removal efficiency of TN (84.72%). During the whole experiment, neither NH_2_OH nor NO_3_^–^-N accumulation was observed, whereas 0.05 mg/L NO_2_^–^-N was observed at 6 h, and then NO_2_^–^-N was exhausted after cultivation for 12 h. This phenomenon was in contrast to the fact that 2.56 mg/L NO_3_^–^-N, instead of NO_2_^–^-N, was detected during the nitrification process by strain EM-B2 (He et al., 2022). Moreover, pH exhibited an upward trend (7.18 → 9.00), suggesting that denitrification with NO_2_^–^-N as a nitrogen source could also be conducted when EN-J1 performed nitrification with NH_4_^+^-N nitrogen alone. This phenomenon was the same as that observed in a study of strain EN-F2, in which 0.11 mg/L NO_2_^–^-N was measured after 6 h of reaction (Zhang et al., 2022). Overall, strain EN-J1 displayed excellent NH_4_^+^-N and TN conversion rates without the accumulation of intermediates and efficiently transformed a large amount of NH_4_^+^-N to gaseous nitrogen. The short NH_4_^+^-N oxidation pathway by strain EN-J1 was beneficial for the thorough treatment of NH_4_^+^-N-contaminated wastewater.

Few bacterial strains with the capacity for the removal of NH_2_OH (one of the intermediate products of nitrification) have been reported, which limits the comprehensive evaluation of the nitrification ability of various strains. Accordingly, NH_2_OH was selected as the only form of nitrogen for further assessment of the NH_2_OH oxidation capacity by EN-J1. Within the initial 6 h of inoculation, a decreasing trend of NH_2_OH was not observed (Figure 3B). After reaction for 18 h, 11.30 mg/L NH_2_OH was consumed from a 22.73 mg/L initial content. Strain EN-J1 consumed 99.96% of the NH_2_OH when the reaction time was extended to 30 h. The maximum elimination rate of NH_2_OH reached 1.22 mg/L/h from 18−24 h, which was lower than the value of 2.12 mg/L/h for *Pseudomonas taiwanensis* EN-F2 (Zhang et al., 2022). Nonetheless, on the basis of comparison with the values for most reported HN-AD strains that can remove NH_2_OH, such as 0.21 mg/L/h for *Glutamicibacter arilaitensis* EM-H8 (Chen et al., 2022) and 0.70 mmol/L/h for *Photobacterium sp*. NNA4 (Liu et al., 2019), strain EN-J1 was more efficient degrading NH_2_OH. Throughout the experiment, the pH and OD_600_ values remained basically stable, and TN slightly decreased from 22.44 to 20.66 mg/L. Furthermore, NO_3_^–^-N (4.06 mg/L) and NH_4_^+^-N (1.86 mg/L) accumulation was observed. Moreover, NO_2_^–^-N production reached the highest value of 13.62 mg/L at 30 h, indicating that strain EN-J1 converted most of the NH_2_OH to NO_2_^–^-N instead of gaseous nitrogen. This phenomenon was consistent with observations of the strain *Pseudomonas putida* Y-9 (Huang et al., 2019) but distinct from those of *Acinetobacter calcoaceticus* HNR (Zhao et al., 2010).

### 3.3. Traits of strain EN-J1 with respect to NO_3_^–^-N and NO_2_^–^-N removal

**FIGURE 4:**
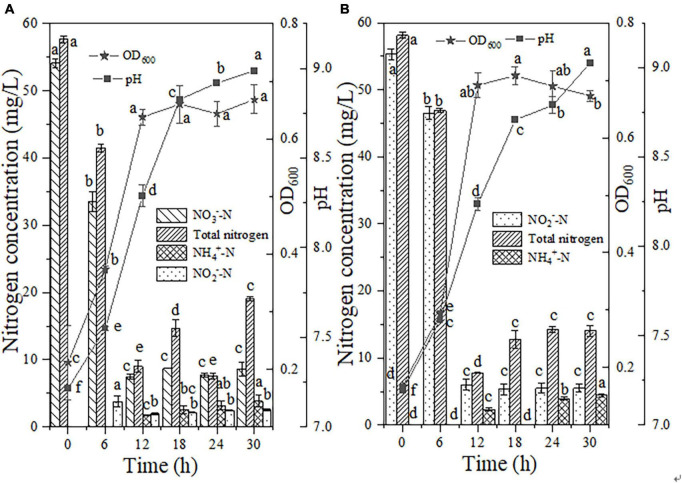
The aerobic denitrification characteristics of strain EN-J1. **(A)** Nitrate (NO_3_^–^-N); **(B)** nitrite (NO_2_^–^-N). Different letters represent significant differences between treatments (*p* < 0.05).

The utilization of NO_3_^–^-N (50 mg/L) by strain EN-J1 under aerobic conditions was explored to illustrate the denitrification characteristics. A conspicuous increase in OD_600_ (0.21 → 0.67) and a decrease in NO_3_^–^-N concentration were observed after 12 h of incubation (Figure 4A). A total of 84.01% of NO_3_^–^-N was reduced with a maximum rate of 4.34 mg/L/h between 6 and 12 h, which was faster than the rates of 1.71 mg/L/h (for 100 mg/L initial NO_3_^–^-N) or 1.90 mg/L/h (for 50 mg/L initial NO_3_^–^-N) observed for *Streptomyces mediolani* EM-B2 (He et al., 2021, 2022) and 1.99 mg/L/h observed for *P. tolaasii* Y-11 (He et al., 2016) but slightly lower than the rates of 4.54 and 5.80 mg/L/h obtained for *Pseudomonas mendocina* X49 (Xie et al., 2021) and *Pseudomonas taiwanensis* EN-F2 (Zhang et al., 2022), respectively. It is worth noting that the degradation efficiency and rate of NO_3_^–^-N were much lower than those of NH_4_^+^-N, indicating that for strain EN-J1, nitrification with NH_4_^+^-N as a nitrogen source was stronger than denitrification with NO_3_^–^-N-only nitrogen. This conclusion was similar to that reported for *Pseudomonas mendocina* X49 (Xie et al., 2021). Furthermore, the TN content decreased (57.64 → 19.07 mg/L), corresponding to a maximum conversion efficiency and rate of 66.92% and 5.41 mg/L/h, respectively. The pH value increased to 8.99. A low level of NO_2_^–^-N (2.54 mg/L) was detected at 30 h, which was different from the result for the strain *Acinetobacter sp*. ND7 (Xia et al., 2020), which exhibited no NO_2_^–^-N accumulation. Approximately 3.84 mg/L NH_4_^+^-N accumulated, which might originate from the disintegration of dead cells. All the results in this section showed that strain EN-J1 could carry out denitrification with a nitrogen source of NO_3_^–^-N and transform most of the NO_3_^–^-N into gaseous nitrogen.

When equivalent amounts of NO_2_^–^-N instead of NO_3_^–^-N were added to the medium, the reduction of NO_2_^–^-N and accumulation of intermediates could be used to further reflect the denitrification characteristics of strain EN-J1. Figure 4B shows that 89.10% of NO_2_^–^-N with an initial concentration of 55.32 mg/L was removed at a maximum degradation rate of 6.75 mg/L/h after 12 h of reaction. The OD_600_ value reached 0.69. The high nitrogen removal rate and cell growth contradicted the conclusion that a high concentration of NO_2_^–^-N inhibits cell growth and denitrification (Wan et al., 2011). After 18 h of cultivation, the removal efficiency of NO_2_^–^-N remained at 90.09%, and the cells reached the stationary phase. The incomplete removal of NO_2_^–^-N further indicated that the heterotrophic nitrification capacity of EN-J1 was superior to its aerobic denitrification capacity, which was also found for the strain *Pseudomonas taiwanensis* EN-F2 (Zhang et al., 2022). Fortunately, the conversion rate (6.75 mg/L/h) was noticeably higher than that of previous HN-AD strains for NO_2_^–^-N removal, such as 3.25 mg/L/h of *Pseudomonas putida* Y-12 (Ye et al., 2017), 1.74 mg/L/h of *Pseudomonas tolaasii* Y-11 (He et al., 2016) and 4.12 mg/L/h of *Ochrobactrum anthropic* LJ81 (Lei et al., 2019). Moreover, 86.84% of TN was transformed with a maximum rate of 6.52 mg/L/h, which was a much higher rate than that of the NO_2_^–^-N-removing strains mentioned above. In addition, during the reduction of NO_2_^–^-N, the level of NH_4_^+^-N increased to the highest value of 4.42 mg/L at 30 h, which decreased the removal efficiency of TN (86.84% → 75.88%). Similar to a report on the strain *Streptomyces mediolani* EM-B2 (He et al., 2021), NO_3_^–^-N could not be detected in this study. The pH increased from 7.19 to 9.03 based on alkali production from NO_2_^–^-N reduction, which further confirmed that strain EN-J1 could efficiently execute denitrification.

### 3.4. Effects of NH_2_OH on the HN-AD process

To investigate the effects of NH_2_OH on the HN-AD process and bacterial cells of strain EN-J1, approximately 10 mg/L NH_2_OH hydrochloride was added to individual samples of nitrogenous media containing NO_3_^–^-N, NO_2_^–^-N, and NH_4_^+^-N.

**FIGURE 5:**
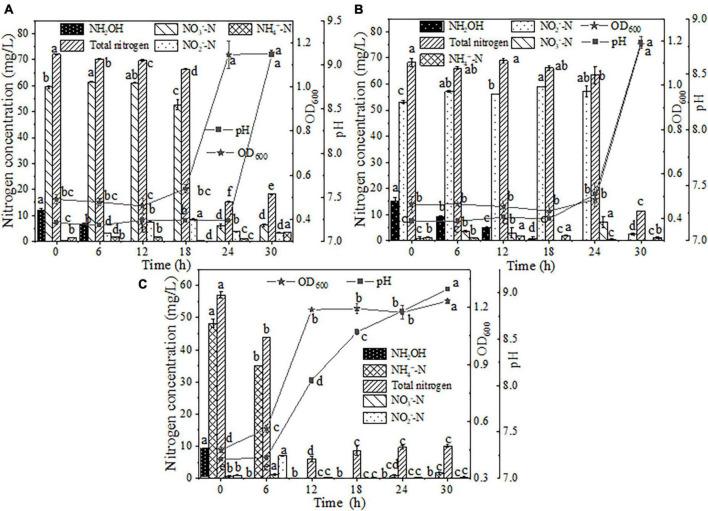
The effects of hydroxylamine (NH_2_OH) on HN-AD. **(A)** Nitrate (NO_3_^–^-N); **(B)** nitrite (NO_2_^–^-N); **(C)** ammonium (NH_4_^+^-N). Different letters represent significant differences between treatments (*p* < 0.05).

The intermediate product accumulation and cell growth values obtained with the addition of NH_2_OH to NO_3_^–^-N medium are shown in Figure 5A. During the initial 12 h of inoculation, 11.93 mg/L NH_2_OH was consumed with an oxidation efficiency of 100.00%. The maximum NH_2_OH consumption rate was 1.11 mg/L/h, which was similar to the result obtained from the NH_2_OH-only removal system (1.22 mg/L/h). The above results signified that the presence of NO_3_^–^-N had almost no influence on NH_2_OH removal. The NO_3_^–^-N concentration slightly increased by 2.07 mg/L within 12 h due to NH_2_OH oxidation, and then decreased promptly to 6.20 mg/L after NH_2_OH was completely depleted, which implied that nitrification with NH_2_OH as a nitrogen source was preferentially performed by strain EN-J1. Within 30 h of inoculation, NO_3_^–^-N consumption reached 89.56%, and a degradation rate of 7.78 mg/L/h was observed, which was markedly superior to the value of 4.34 mg/L/h obtained with the NO_3_^–^-N-only reduction system. Concurrently, the cell growth of strain EN-J1 ranged from 0.49 to 1.15, which was also higher than that in the NO_3_^–^-N-only reaction system. It could be concluded that when NH_2_OH was replenished, the maximum rate of NO_3_^–^-N was increased by 3.44 mg/L/h, and the OD_600_ value was boosted by 0.48. The positive effect of NH_2_OH on NO_3_^–^-N removal was consistent with the finding that a low dosage of NH_2_OH promoted NO_3_^–^-N conversion (Zhang et al., 2020). With the consumption of NH_2_OH and NO_3_^–^-N, the pH increased to 9.11. The TN content exhibited a conspicuous reduction from 72.09 to 18.20 mg/L, and the corresponding conversion maximum efficiency and rate reached 74.75% and 8.50 mg/L/h, respectively. The high TN removal rate was also prominently higher than that in the sole NO_3_^–^-N reduction system (5.41 mg/L/h) and the system with NH_2_OH alone (0.60 mg/L/h). Throughout the experiment, only 3.26 mg/L NO_2_^–^-N and 3.53 mg/L NH_4_^+^-N accumulation was observed. All results indicated that NH_2_OH exhibited a position effect on NO_3_^–^-N and TN removal.

When NH_2_OH was added to NO_2_^–^-N medium, a stagnation period of strain EN-J1 was observed and the OD_600_ value remained at approximately 0.45 within 24 h of cultivation (Figure 5B) but then rapidly increased to 1.18 at 30 h. Cell growth was boosted by 0.51 compared to that in the only NO_2_^–^-N removal system. The pH increased from 7.18 to 8.79. During this period, the degradation efficiency and maximum removal rate of NH_2_OH reached 100.00% and 1.01 mg/L/h, respectively, at an initial concentration of 15.14 mg/L. This rate was slightly lower than that of the NH_2_OH only system (1.22 mg/L/h), which indicated that NO_2_^–^-N could delay the consumption of NH_2_OH. Nonetheless, NO_2_^–^-N exhibited no influence on NH_2_OH conversion efficiency, which was the same as the result for *Pseudomonas taiwanensis* EN-F2 (Zhang et al., 2022). Similar to the phenomenon in the case of mixed NH_2_OH and NO_3_^–^-N, NO_2_^–^-N increased by approximately 4.00 mg/L because of NH_2_OH oxidation within 24 h and then declined to 2.54 mg/L after NH_2_OH depletion. The degradation efficiency and maximum degradation rate of NO_2_^–^-N reached 95.22% and 9.11 mg/L/h, respectively. Markedly, the NO_2_^–^-N reduction rate (9.11 mg/L/h) was higher than that (6.75 mg/L/h) of the NO_2_^–^-N-only nitrogen degradation system after NH_2_OH addition. The NO_2_^–^-N conversion rate was improved by 2.36 mg/L/h because of the addition of NH_2_OH, which was opposite to a report that NO_2_^–^-N consumption was inhibited by NH_2_OH (Noophan et al., 2004). During the nitrogen removal process, only 1.06 mg/L NH_4_^+^-N, rather than NO_3_^–^-N, accumulated. The maximum TN consumption rate reached 8.69 mg/L/h, and this value was enhanced by 2.17 mg/L/h after NH_2_OH was added. All of the above phenomena indicated that the presence of NH_2_OH could promote cell growth and NO_2_^–^-N and TN removal rates.

Similarly, NH_2_OH (approximately 10 mg/L) as a supplementary substance was added to the NH_4_^+^-N-containing medium. Strain EN-J1 proliferated rapidly after 6 h of inoculation and entered the logarithmic phase (Figure 5C). The peak value of OD_600_ reached 1.23 in the mixed media, which was substantially higher than that (0.87) in the NH_4_^+^-N-only treatment system. The OD_600_ value was boosted by 0.36 after NH_2_OH addition, which was also found by Yu et al. (2019). NH_2_OH was exhausted, and a maximum rate of 1.54 mg/L/h was achieved. This result indicated that NH_4_^+^-N played a positive role in the NH_2_OH removal process. The NH_4_^+^-N content started to drop from 48.19 to 35.11 mg/L in the first 6 h, and then it took another 6 h to completely remove the remaining 35.11 mg/L NH_4_^+^-N, corresponding to a maximum degradation rate of 5.85 mg/L/h. The addition of NH_2_OH lowered the removal rate of NH_4_^+^-N compared with that (7.12 mg/L/h) for NH_4_^+^-N nitrogen biodegradation alone, which implied that NH_2_OH retarded NH_4_^+^-N conversion. Even so, NH_4_^+^-N was still completely removed at 12 h. The content of NO_3_^–^-N correspondingly dropped from 0.53 to 0 mg/L. NO_2_^–^-N accumulated to the highest value of 7.06 mg/L at 6 h and then dropped to 0.28 mg/L. The pH increased to 9.04, which suggested that strain EN-J1 performed denitrification during the nitrification process. In addition, 82.34% of TN was removed at a maximum rate of 6.34 mg/L/h, which was close to the rate of 6.42 mg/L/h obtained with NH_4_^+^-N as the only nitrogen source. These results indicated that TN removal was not affected by NH_2_OH, which was inconsistent with a study showing that low levels of NH_2_OH could promote TN removal efficiency (Zekker et al., 2012). Overall, the conversion efficiencies of NH_4_^+^-N and TN were not affected by NH_2_OH addition.

In summary, when a low level of NH_2_OH (10.00 mg/L) was supplemented to NO_3_^–^-N or NO_2_^–^-N media as a mixed nitrogen source, the cell growth of strain EN-J1 was boosted by 0.48 and 0.51, respectively. Synchronously, the degradation rates of NO_3_^–^-N and NO_2_^–^-N were enhanced by 3.44 and 2.36 mg/L/h, and the corresponding TN degradation rates were enhanced by 3.09 and 2.17 mg/L/h, respectively. However, the presence of NH_2_OH hydrochloride had no effect on NH_4_^+^-N removal efficiency.

### 3.5. The effects of NO_2_^–^-N on NO_3_^–^-N/NH_4_^+^-N removal

**FIGURE 6:**
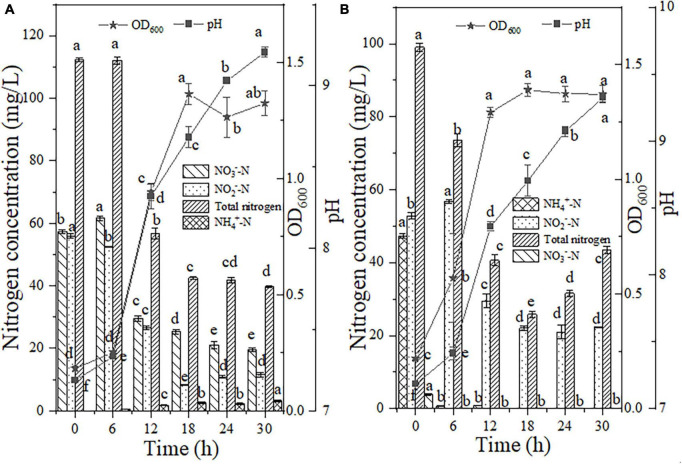
The effects of nitrite (NO_2_^–^-N) on HN-AD. **(A)** Nitrate (NO_3_^–^-N); **(B)** ammonium (NH_4_^+^-N). Different letters represent significant differences between treatments (*p* < 0.05).

The bacterial cell adaptation and nitrogen consumption performance after the addition of a high dosage of NO_2_^–^-N (55.97 mg/L) to the NO_3_^–^-N reaction medium are shown in Figure 6A. Within 6 h of the reaction, the NO_2_^–^-N content decreased by 3.51 mg/L in the presence of strain EN-J1. The degradation rates of NO_2_^–^-N and NO_3_^–^-N reached individual peak values of 4.31 and 5.34 mg/L/h between 6 and 12 h. Compared with that in the NO_3_^–^-N-only treatment system, the NO_3_^–^-N reduction rate was enhanced by 1.0 mg/L/h after NO_2_^–^-N addition, which demonstrated that the presence of NO_2_^–^-N significantly promoted the consumption of NO_3_^–^-N. This result was opposite to a report on the strain *Pseudomonas taiwanensis* EN-F2 (Zhang et al., 2022), in which the NO_3_^–^-N removal rate was limited by the addition of NO_2_^–^-N. However, NO_3_^–^-N exhibited an inhibitory effect on NO_2_^–^-N, which was consistent with the result for *Thauera sp*. SND5 (Wang and He, 2020). With nitrogen elimination, strain EN-J1 grew rapidly, and the OD_600_ value reached a peak of 1.32 after inoculation for 30 h. The pH showed an upward trend from 7.19 to 9.21. A total of 64.52% of the high concentration of TN was consumed with the highest rate of 9.25 mg/L/h, which was notably faster than that in the NO_3_^–^-N-only (5.41 mg/L/h) and NO_2_^–^-N-only (6.52 mg/L/h) treatment systems. During the whole experiment, only a low dosage of NH_4_^+^-N (3.12 mg/L) accumulated. These results showed that cell growth and the maximum conversion rates of NO_3_^–^-N and TN were enhanced by supplementation with NO_2_^–^-N. This conclusion was contradictory to most previous reports that NO_2_^–^-N inhibited the nitrogen removal process.

High proportions of NO_2_^–^-N and NH_4_^+^-N were mixed as nitrogen sources to reveal the effect of NO_2_^–^-N on the HN process. In this mixed nitrogen reaction system, NH_4_^+^-N conversion was performed with an efficiency of 98.50% in the first 6 h (Figure 6B). During NH_4_^+^-N degradation, the NO_2_^–^-N content was enhanced by 4.00 mg/L with an increase in cell growth from 0.22 to 0.57, implying that NH_4_^+^-N was preferentially converted to NO_2_^–^-N, which was different from the report that NH_4_^+^-N and NO_2_^–^-N could be degraded together by *Streptomyces mediolani* EM-B2 (He et al., 2022). The increase in and disappearance of NO_2_^–^-N (4.00 mg/L) implied that NH_4_^+^-N was oxidized through the pathway NH_4_^+^-N → NO_2_^–^-N → nitrogenous gas. After 18 h of reaction, NH_4_^+^-N was exhausted, corresponding to the highest rate of 7.77 mg/L/h. The rate of NH_4_^+^-N degradation in this period was slightly enhanced by 0.65 mg/L/h after NO_2_^–^-N addition, which was similar to the result for *Ochrobactrum anthropic* LJ81 (Lei et al., 2019). The NO_2_^–^-N content was greatly reduced to 29.54 mg/L and the corresponding maximum degradation rate of 4.54 mg/L/h was detected between 6 and 12 h. When compared with the NO_2_^–^-N-only removal system (6.75 mg/L/h), the rate in mixed media was lower. An inhibitory effect on NO_2_^–^-N consumption was shown in the presence of NH_4_^+^-N, which was in contrast to the results for strain *Arthrobacter arilaitensis* Y-10 reported by He et al. (2017). Strain EN-J1 did not produce NO_3_^–^-N when cultured with mixed NO_2_^–^-N and NH_4_^+^-N. The pH increased (7.18 → 9.32) with nitrogen consumption. Moreover, at an initial TN content of 99.05 mg/L, 56.15% was removed with a maximum rate of 5.49 mg/L/h, which was slower than that of NH_4_^+^-N (6.42 mg/L/h) or NO_2_^–^-N (6.51 mg/L/h) alone. All the above findings demonstrated that although the removal rate of TN was not enhanced, NH_4_^+^-N removal and cell growth were promoted by NO_2_^–^-N addition.

In summary, after the addition of 50 mg/L NO_2_^–^-N, the conversion rates of NO_3_^–^-N and NH_4_^+^-N increased by 1.00 and 0.65 mg/L/h, respectively, and the corresponding cell growth increased by 0.65 and 0.50, respectively. Therefore, the additional addition of NH_2_OH and NO_2_^–^-N promoted the conversion rates of inorganic nitrogen species, which has great advantages for thorough wastewater treatment. However, deeper investigation of strain EN-J1 needs to be performed, especially regarding its potential in real wastewater treatment.

### 3.6. Nitrogen balance analysis of HN-AD

A nitrogen balance analysis of NH_4_^+^-N, NO_2_^–^-N, NH_2_OH, and NO_3_^–^-N in the current (bacterial strain EN-J1) system was calculated and is described in Table 1. The initial level of TN in the NH_2_OH-only system was 27.44 mg/L, and the NH_4_^+^-N, NO_3_^–^-N, and NO_2_^–^-N levels were approximately 54 mg/L. After 12 h of continuous reaction, 75.59% of the initial NH_4_^+^-N was lost, implying that the formation of gaseous nitrogen was the main route of NH_4_^+^-N removal by strain EN-J1. More importantly, the TN loss efficiency was considerably higher than the values of 40.20% for *Acinetobacter calcoaceticus* HNR (Zhao et al., 2010) and 27.11% for *Exiguobacterium mexicanum* SND-01 (Cui et al., 2021). During NH_4_^+^-N oxidation, intracellular-N (8.37 ± 0.70) and organic-N (4.34 ± 0.49) were detected, whereas the accumulation of NH_2_OH, NO_2_^–^-N, and NO_3_^–^-N was not observed, which was the same as the results in the NH_4_^+^-N-only nitrogen oxidation system. NO_2_^–^-N was detected at 6 h and was then exhausted at 12 h when NH_4_^+^-N was selected as a single nitrogen source, which further demonstrated that the oxidation route of NH_4_^+^-N was NH_4_^+^-N → NO_2_^–^-N → gaseous nitrogen. For NH_2_OH, 7.18% nitrogen loss was detected, and most NH_2_OH was converted to other nitrogen forms after cultivation for 30 h, in which 1.43 mg/L NH_4_^+^-N, 11.56 mg/L NO_2_^–^-N, and 1.62 mg/L NO_3_^–^-N were detected. These results were inconsistent with reports that NO_2_^–^-N was not observed with *Glutamicibacter arilaitensis* EM-H8 (Chen et al., 2022) and *Alcaligenes faecalis* NR (Zhao et al., 2012). This accumulation of intermediate products further proved that the pathway for NH_2_OH conversion was NH_2_OH → NO_2_^–^-N → NO_3_^–^-N → gaseous nitrogen.

**TABLE 1:** Nitrogen balance during HN-AD process.

Substance	Initial TN (mg/L)	Final N (mg/L)					Intracellular-N (mg/L)	N lose (%)
		**NH_4_^+^-N**	**NH_2_OH**	**NO_2_^–^-N**	**NO_3_^–^-N**	**Organic-N**		
NH_4_^+^-N	52.07 ± 0.36	0	0	0	0	4.34 ± 0.49	8.37 ± 0.70	75.59 ± 0.03
NH_2_OH	27.44 ± 0.69	1.43 ± 0.10	4.71 ± 0.23	11.56 ± 0.37	1.62 ± 0.68	3.01 ± 0.41	3.14 ± 0.58	7.18 ± 0.25
NO_3_^–^-N	54.92 ± 0.45	1.28 ± 0.14	0	1.99 ± 0.40	7.27 ± 0.45	4.79 ± 0.64	7.85 ± 0.41	57.79 ± 0.55
NO_2_^–^-N	54.21 ± 0.73	2.42 ± 0.12	0	6.13 ± 0.13	0	3.16 ± 0.23	7.67 ± 0.81	64.25 ± 0.38

In addition, approximately 57.79% of the initial TN was lost, and 8.72% of it was converted to organic-N when strain EN-J1 was cultivated with NO_3_^–^-N medium for 18 h, which illustrated that NO_3_^–^-N was almost completely reduced into gaseous nitrogen. The nitrogen loss from NO_3_^–^-N in the initial TN was higher than that of *Pseudomonas mendocina* LYX (51.90%) (Li et al., 2020) and *Acinetobacter sp.* YT03 (28.33%) (Li et al., 2019). On the basis of the detection of intermediate products, low concentrations of NH_4_^+^-N (1.28 mg/L) and NO_2_^–^-N (1.99 mg/L) accumulated, which further confirmed that the NO_3_^–^-N reduction pathway was NO_3_^–^-N → NO_2_^–^-N → gaseous nitrogen. Similarly, strain EN-J1 could transform most NO_2_^–^-N into gaseous nitrogen, and 64.25% nitrogen loss was achieved. This was markedly higher than the result of 38.88% for *Streptomyces mediolani* EM-B2 (He et al., 2022). To date, few studies have reported that HN-AD strains have the capacity to convert large amounts of NO_2_^–^-N into gas. Moreover, 3.16 mg/L organic-N and 2.42 mg/L NH_4_^+^-N were detected. According to the above results, the removal route of NO_2_^–^-N could be inferred to be NO_2_^–^-N → gaseous nitrogen. All of the above observations illustrated that NH_4_^+^-N, NO_3_^–^-N, and NO_2_^–^-N were mainly converted into gaseous nitrogen by strain EN-J1 to efficiently implement the HN-AD process.

### 3.7. Enzyme activity analysis

According to previous reports, the enzyme activities of AMO, HAO, NR, and NIR are related to the oxidation of NH_4_^+^-N and NH_2_OH and the reduction of NO_3_^–^-N and NO_2_^–^-N, respectively, (Ren et al., 2014). Therefore, the successful detection of related enzymes could verify the HN-AD pathway for bacteria. To date, no reports have studied HN-AD-related enzyme activities in *A. johnsonii.* In this study, the specific activities of AMO, HAO, NR, and NIR were successfully detected as 0.54, 0.15, 0.14, and 0.01 U/mg protein, respectively, (Table 2). By comparison, AMO exhibited the highest specific activity, which further confirmed the maximum conversion rate (7.12 mg/L/h) of NH_4_^+^-N. More importantly, the specific activities of these enzymes in strain EN-J1 were notably stronger than those in previously studied bacteria. For example, although the NR activity of strain EM-B2 (0.12 U/mg protein) was similar to that (0.15 U/mg protein) of EN-J1, the activities of both AMO (0.43 U/mg protein) and NIR (0.01 U/mg protein) were significantly lower than those of EN-J1 (He et al., 2022). Lower activities of HAO and NR in *Pseudomonas taiwanensis* J488 were detected, with values of 0.05 and 0.09 U/mg protein (He et al., 2020). HAO and NR activities of only 0.04 and 0.02 U/mg protein were obtained for *Pseudomonas putida* NP5 (Yang et al., 2019), and AMO, HAO, and NR activities of 0.05, 0.05, and 0.02 U/mg protein were obtained for *Halomonas salifodinae* (Hu et al., 2021). The high enzyme activities of AMO, HAO, NR, and NIR further verified the excellent HN-AD performance of strain EN-J1.

**TABLE 2:** Related specific activities of HN-AD.

Enzymes	Specific activities (U/mg)
Ammonia monooxygenase (AMO)	0.54 ± 0.06
Hydroxylamine oxidoreductase (HAO)	0.15 ± 0.01
Nitrate reductase (NR)	0.14 ± 0.13
Nitrite reductase (NIR)	0.01 ± 0.02

## 4. Conclusion

*Acinetobacter johnsonii* EN-J1 presented an outstanding nitrogen conversion capacity. The maximum conversion rates of NH_4_^+^-N, NH_2_OH, NO_3_^–^-N, and NO_2_^–^-N reached 7.12, 1.22, 4.34, and 6.75 mg/L/h, respectively. The addition of NH_2_OH and NO_2_^–^-N enhanced the HN-AD performance of strain EN-J1. The removal rates of NO_3_^–^-N and NO_2_^–^-N were increased by 3.44 and 2.36 mg/L/h, respectively, after the addition of 10 mg/L NH_2_OH, while the removal rates of NO_3_^–^-N and NH_4_^+^-N were increased by 1.00 and 0.65 mg/L/h under 50 mg/L NO_2_^–^-N addition. The enzymes involved in HN-AD were measured at 0.54, 0.15, 0.14, and 0.01 U/mg protein. The nitrogen balance results revealed that more than 55.00% of the initial TN was converted to gaseous nitrogen when the wastewater contained NH_4_^+^-N, NO_3_^–^-N, or NO_2_^–^-N. Altogether, strain EN-J1 exhibits potential for application in the treatment of mixed nitrogen-polluted wastewater.

## Data availability statement

The raw data supporting the conclusions of this article will be made available by the authors, without undue reservation.

## Author contributions

MZ: experimental design, data calculation, figures construction, manuscript writing, and experimental operation. TH: investigation, experimental idea, experimental design, experimental operation, data calculation, figures construction, manuscript revising, and fund investment. QW: experimental operation and investigation. MC: experimental operation. All authors contributed to the article and approved the submitted version.
